# Application of Precision Medicine in Neurodegenerative Diseases

**DOI:** 10.3389/fneur.2018.00701

**Published:** 2018-08-23

**Authors:** Claudia Strafella, Valerio Caputo, Maria R. Galota, Stefania Zampatti, Gianluca Marella, Silvestro Mauriello, Raffaella Cascella, Emiliano Giardina

**Affiliations:** ^1^Department of Biomedicine and Prevention, Tor Vergata University, Rome, Italy; ^2^Emotest Laboratory, Pozzuoli, Italy; ^3^Molecular Genetics Laboratory UILDM, Santa Lucia Foundation, Rome, Italy; ^4^Section of Legal Medicine, Tor Vergata University, Rome, Italy; ^5^Department of Chemical-Toxicological and Pharmacological Evaluation of Drugs, Catholic University Our Lady of Good Counsel, Tirana, Albania

**Keywords:** neurodegenerative diseases, precision medicine, research networks, Parkinson disease, omic profiles

## Abstract

One of the main challenges for healthcare systems is the increasing prevalence of neurodegenerative pathologies together with the rapidly aging populations. The enormous progresses made in the field of biomedical research and informatics have been crucial for improving the knowledge of how genes, epigenetic modifications, aging, nutrition, drugs and microbiome impact health and disease. In fact, the availability of high technology and computational facilities for large-scale analysis enabled a deeper investigation of neurodegenerative disorders, providing a more comprehensive overview of disease and encouraging the development of a precision medicine approach for these pathologies. On this subject, the creation of collaborative networks among medical centers, research institutes and highly qualified specialists can be decisive for moving the precision medicine from the bench to the bedside. To this purpose, the present review has been thought to discuss the main components which may be part of precise and personalized treatment programs applied to neurodegenerative disorders. Parkinson Disease will be taken as an example to understand how precision medicine approach can be clinically useful and provide substantial benefit to patients. In this perspective, the realization of web-based networks can be decisive for the implementation of precision medicine strategies across different specialized centers as well as for supporting clinical/therapeutical decisions and promoting a more preventive and participative medicine for neurodegenerative disorders. These collaborative networks are essentially addressed to find innovative, sustainable and effective strategies able to provide optimal and safer therapies, discriminate at risk individuals, identify patients at preclinical or early stage of disease, set-up individualized and preventative strategies for improving prognosis and patient's quality of life.

## Introduction

One of the major consequences of rapidly aging populations is the increasing burden of neurodegenerative disorders and, subsequently, the higher mortality/morbidity rates and healthcare costs for treatment, hospitalization and care assistance. To date, about 16% of people is over 65 in Europe and they are expected to rise to 25% by 2030^1^. Neurodegenerative disorders include a large spectrum of age-related neurological pathologies characterized by the progressive^1^ loss or dysfunction of neurons in specific areas of the brain and/or of the spinal cord. Patients affected with this kind of disorders display variable clinical features, including cognitive decline, speech difficulties and motor impairment (1). Among them, the most prevalent neurodegenerative disorders are dementias which affect approximately 7 million people in Europe and are estimated to double by 2040. The average duration of disease ranges between 2 and 10 years, during which patients need special care and therapies (if they are available) to face their disabilities. The overall cost for treating patients suffering from neurodegenerative diseases is approximately **€**130 billion/year^1^. In this perspective, the increasing prevalence of such pathologies together with the rapidly aging populations represent a real challenge for healthcare systems of any country and the overall society. The enormous progresses made in the field of biomedical research and the application of artificial intelligence systems have been crucial to understand how genes, epigenetic modifications aging, nutrition, drugs, microbiome and other environmental factors can impact health and disease. Regarding neurodegenerative diseases, Alzheimer (AD) and Parkinson disease (PD) are the mostly investigated pathologies, as shown by the several dedicated database and research programs (JPND research program, MDS gene, PDGene, AlzGene)^1^^,^^2^. However, most of the up-to-date informations increased knowledge about the existence of Mendelian familial forms of AD and PD caused by single gene mutations and complex forms of disease characterized by several genetic polymorphisms, which contribute to the susceptibility to AD and PD in combination with non-genetic factors. Currently, scientific research can rely on the availability of high-technology and computational tools for large-scale analysis. Therefore, neurodegenerative disorders can be investigated at a deeper level, providing a more comprehensive overview of disease and paving the way for the application of precision medicine to these pathologies. On this subject, the creation of collaborative networks including medical centers, research institutes and highly skilled specialists can be decisive for moving precision medicine from bench to bedside. These collaborative networks should pull together to find innovative, sustainable and effective pathways aiming to: (i) provide optimal and safer therapies (based on pharmacogenetics approaches), (ii) discriminate at-risk individuals, (iii) identify patients at the preclinical or the earliest stages of disease, (iv) set-up individualized and preventative strategies for improving prognosis and patient's quality of life. In this context, the present review will discuss the main components that could be considered for developing precise and personalized treatments for neurodegenerative disorders. To this purpose, special attention will be given to PD since it represents an excellent example to show how precision medicine can be clinical useful and provide substantial benefit to patients.

## Overview on parkinson's disease

Parkinson's disease (PD) is a progressive chronic neurodegenerative disorder caused by the loss of dopaminergic neurons in the Substantia Nigra *pars Compacta* (SNc) and the formation of Lewy bodies (abnormal aggregates of protein) (1). It affects more than 10 million people worldwide, with a variable prevalence depending on age, male sex and geographic position (2). PD dramatically impacts the life of patients as well as the overall health system. In US, the costs for the management and treatment of PD patients are $14.4 billion/year and are estimated to double by 2040. In Italy these expenses are approximately **€**8.340/patient (3).

Clinical hallmarks of PD are resting tremor, rigidity, bradykinesia, and postural instability, although patients can also experience non-motor symptoms, such as neuropsychiatric, olfactory and sleep disturbances (1, 4). The disease can present an idiopathic and a vascular form. The former is commonly sporadic, except for 10% of cases showing a familial segregation (5). The latter is mostly observed among the elderly, in combination with hypertension, hyperlipidemia and diabetes (6). The etiopathogenesis of idiopathic PD involves a complex interplay between environmental and genetic factors (7). Among environmental factors, cigarette smoking, coffee and tea consumption, pesticides, drug use (statins, anti-hypertensive, anti-inflammatory drugs), comorbidities (diabetes, anemia, anxiety, depression) have been shown to influence the onset and course of PD (2, 7–9). The genetic contribution to familial and complex PD has been extensively studied. Families with monogenic familial forms of PD often carry mutations in *SNCA* (4q22.1, α*-synuclein*) gene (Table 1). It is one of the major contributors to disease and encodes for α-synuclein, a protein involved in biogenesis and trafficking of synaptic vesicles, dopamine metabolic process, apoptosis, histone acetylation and microtubule polymerization (10). *LRRK2* (12q12, *Leucine rich repeat kinase 2*) accounts for the 4% of familial PD and it is involved in the regulation of autophagy, synaptic vesicle trafficking cytoskeleton dynamics and neuroinflammation (8). *VPS35* (16q11.2, *Vacuolar protein sorting 35*) is implicated in retrograde vesicle transport and accounts for 1% of hereditary PD (10). These genes are responsible for autosomal dominant hereditary patterns, whereas *PARK2* (6q26, *Parkin*), *PINK1* (1p36.12, *PTEN-induced putative kinase 1*) and *DJ1* (1p36.23, *Protein Deglycase DJ-1*) cause autosomal recessive forms: *PARK2* is the most frequent cause of early-onset autosomal recessive PD and it is implicated in mytophagy (8, 10). *PINK1* is the second most frequently mutated gene of early-onset autosomal recessive PD and it is known to interact with *PARK2*. *DJ1* is also responsible for early-onset forms of PD and it interacts with *PARK2* and *PINK1* (Table 1) (10). Furthermore, variants in several genes have been found to increase the risk of PD. In particular, a meta-analysis of five GWAS (Genome-Wide Association Studies) reported eleven associated-loci, including SNPs of *SNCA, LRRK2, GBA* (1q22, *Glucosidase* β), and *HLA-DRB5* (6p21.32, *Major histocompatibility complex, class II, DR* β*-5*) (4, 9–11).

**Table 1:** List of the most common mutations found in patients affected by Mendelian forms of PD (43).

**Gene**	**Most common mutations**	**Inheritance pattern**	**Clinical phenotype**
*SNCA* (*α-synuclein*)	A53T		AO:46 years, L-Dopa responsive parkinsonism, cognitive decline, autonomic dysfunction, dementia
	A30P	AD	AO:52 years, incomplete penetrance, L-Dopa responsive parkinsonism, cognitive decline, autonomic dysfunction, dementia
	E46K		AO:50-60 years, Lewy body dementia presenting within 2 years of diagnosis
*LRRK2* (*Leucine-Rich Repeat Kinase 2*)	G2019S	AD	AO:60 years, age-dependent penetrance: 28% at 59 years, 51% at 60 years, 74% at 79 years; slow progression and good response to L-Dopa, dementia is rare
	R1441G	AD	AO:40-90 years, highly penetrance: 95% at the age of 75 years, clinical symptoms typical of sporadic PD
*VPS35* (*Vacuolar Protein Sorting 35*)	D620N, P316S, R524W	AD	AO:53 years, incomplete penetrance, bradykinesia, resting tremor, and good response to levodopa therapy
*PARK2* (*Parkin*)	T240R	AR	AO < 40 years, foot dystonia, psychiatric symptoms, poor response to treatment
*PINK1* (*PTEN induced kinase 1*)	G309D, W437X	AR	Idiopathic-like parkinsonism, L-Dopa responsive, slow progression, dystonia, sleep disorders, pyramidal signs, psychiatric co-morbidities (anxiety and depression), no reports of dementia
*DJ-1* (*protein Deglycase DJ-1*)	L166P	AR	AO in the mid-twenties, phenotype is similar to *PARK2/PINK1*-related forms, slow progression, cognitive problems
*ATP13A2* (*ATPase type 13A2*)	F182L, G504R, G877R, T12M, G533R, A746T	AR	Atypical juvenile PD, rapid progression, dementia, dystonia, supranuclear palsy, pyramidal signs, low response to L-Dopa
*PLA2G6* (*Phospholipase A2, group VI*)	R747W	AR	Juvenile onset parkinsonism, dystonia, L-Dopa responsive, iron accumulation in the brain
*FBXO7* (*F-box only protein 7*)	T22M, L34R	AR	Juvenile onset parkinsonism-pyramidal syndrome, early onset spastic paraplegia, later manifestation of dopa-responsive parkinsonism

*Mutations have been classified according to the gene, inheritance pattern and clinical phenotype. AD, autosomal dominant; AR, autosomal recessive; AO, age of onset*.

To date, an effective therapy against PD is unknown. Although PD displays high clinical and pathological heterogeneity, patients can rely on specific drugs to control a wide range of symptoms, including bradykinesia, rigidity and resting tremor. Most of the drugs utilized for the treatment of PD essentially works by enhancing dopaminergic neurotransmission, among which Levodopa (L-Dopa) is the most effective drug to treat motor symptoms in early and advanced stages of PD (8, 12, 13). Except for L-Dopa (precursor of Dopamine), current therapies can be divided into “dopaminergic” and “non-dopaminergic” drugs (14). The first class of drugs includes dopamine receptor agonists (e.g., apomorphine), catechol-O-methyltransferase (COMT, e.g. entacapone) inhibitors and monoamine oxidase (MAO, e.g., selegiline) inhibitors. Dopaminergic drugs are generally prescribed to reduce motor fluctuations in patients with PD. In particular, MAO inhibitors extend the duration of L-Dopa action time, although the occurrence of L-Dopa-Induced Dyskinesia (LID) along with the disease progression represents the major limit of these therapies. Current non-dopaminergic drugs mainly work as antagonists of NMDA (N-methyl-D-aspartate) receptor (such as Amantadine) providing mild benefits and reducing LID. Other non-dopaminergic drugs are still under preclinical and clinical trials (e.g., metabotropic glutamate receptor antagonists) (14). Moreover, small molecule epigenetic modulators targeting DNMTs (DNA methyltransferase), HDACs (Histone deacetylase) and HATs (Histone acetyltransferase) are also undergoing preclinical and clinical trials as novel therapeutic tools for PD (15).

## Risk assessment for neurodegenerative disorders: use of polygenic scores and epistatic interactions

The application of NGS technologies to the study of neurodegenerative disorders provided a more comprehensive picture of the complexity of these pathologies, highlighting the presence of several susceptibility variants with a specific impact on the onset and progression of such disorders. However, the variants detected by NGS approaches explained a relatively small portion of the overall disease susceptibility if they are considered alone, with ORs between 1.1 and 1.5 in most cases. If taken together, instead, small-effect variants can provide substantial insights into the genetic architecture and biological scenery of complex diseases (16–19). This aspect can be particularly useful for the creation of polygenic scores able to configure a reliable and clinically useful risk profile. Polygenic scores exploit high and low effect variants to predict the risk of developing a disease and stratify patients with specific endophenotype and earlier/later age of onset (16). Extensive research has been performed in order to compute a score which is able to estimate the risk for PD. To date, one of the proposed models showed that patients with a polygenic score >1.5 had an earlier age of onset (< 40 years, OR: 4.8) compared to patients with later onset (>80 of age) of PD. In addition, the study suggested that early forms of disease may not only result from rare, highly penetrant mutations (such as homozygous mutations in *PARK2*) but also from the additive effect of more common, small-effect polygenic alleles (e.g., variants in *LRRK2*). Over the age of onset, another study presented a polygenic risk score for PD which came out to be slightly associated (*p* = 0.037; OR: 1.16, CI95%:1.01–1.33) with “nonamnestic” MCI (affecting cognitive domains only) condition (20). Altogether, polygenic scores proved to be excellent tools to stratify subjects at higher risk of neurodegenerative disorders, help clinicians in performing a timely diagnosis, improve the comprehension of gene-gene, gene-environment interactions and the overall susceptibility to disease. Besides polygenic score models, the implementation of a personalized intervention for neurodegenerative disorders should also consider the impact of gene-gene interactions on the disease severity and progression, which are generally known as epistatic effects. In contrast to polygenic scores which calculate the additive effects of independent risk variants across multiple loci, genetic epistasis evaluates the outcome of the interaction between a gene and one or more genes (16). The importance of investigating epistasis lies in the fact that gene-gene interactions strongly contribute to the regulation of biochemical and signaling pathways underlying the etiopathogenesis and the course of complex pathologies, including neurodegenerative disorders. However, epistatic studies are difficult to realize because of the frequent false positive/negative results, low statistical power and the fact that statistical epistasis does not always correspond to the real biological epistasis and vice versa (16). Concerning epistatic mechanisms involved in PD, a positive interactive effect of *GBA* x *LRKK2* (*p* = 0.007) has been found to be associated with PD clinical symptoms. Authors suggested that *GBA* × *LRKK2* interaction may contribute to impair the autophagy activity and thereby enhance the aggregation of α-synuclein in the dopaminergic neurons. However, this epistatic effect has been reported only in a Chinese population and should be therefore replicated in other cohorts (21). Moreover, two significant interactions were observed in a study involving a cohort of US, Dutch and German patients. The authors detected two pairs of interactions associated with the risk of developing PD in Dutch and German populations, which are *UBE2J1* x *GPR107* (*p* = 8.27 ^*^10^−7^) and *DUSP12* × *DOCK4* (*p* = 8.30 ^*^10^−7^). Interestingly, *UBE2J1, DUSP12*, and *DOCK4* are implicated in some of the biological pathways leading to the pathogenesis of PD, including α-synuclein accumulation, neurite differentiation and proteasomal dysfunction. The relationship between *GPR107* and PD remains to be clarified (22).

Finally, the identification of epistatic effects can be very helpful to provide a more comprehensive picture of disease susceptibility as well as to reveal essential components of the etiopathogenetic processes leading to the development of neurodegenerative disorders such as PD. In addition, the greater knowledge of epistatic mechanisms may be helpful to better understand not only gene-gene interaction networks but also their relationship with epigenetic modifications.

## Epigenetics

Growing evidence proved that epigenetic mechanisms play a critical role in neurodegeneration and can be therefore applied for clinical, diagnostic and therapeutic purposes. Generally, “neuroepigenetic modifiers” can act during the fetal life depending on maternal exposure, in the early life during the brain development and in later life, influencing the onset and progression of neurodegenerative disorders. Epigenetic modifications have been extensively recognized as crucial modulators of gene expression thanks to their ability to switch on or off specific genes and changing the chromatin structure in response to aging and environmental (smoking, chemicals, diet) changes. Concerning neurobiological processes and neurodegeneration, most of the studies have been performed on DNA methylation, ncRNA elements and, to a lesser extent, on chromatin remodeling and histone modifications (23). In this review, only ncRNAs will be discussed because they are more likely to have a clinical utility in neurodegenerative disease. Among ncRNAs, miRNAs are certainly the most investigated elements, given their expression in response to several physiological and pathological conditions, their essential role as post-transcriptional regulators and the possibility to detect them in a variety of body fluids (including blood, urine, saliva, tears, breast milk and cerebrospinal fluid -CSF). MiRNAs have been therefore extensively studied because of their potential as biomarkers for diagnosis, prognosis, response to treatment as well as disease-modifying agents (24, 25). In this context, most of the current research aimed to elucidate the role of miRNAs in neurogenesis and neurodegeneration mechanisms. In fact, miRNAs can take part in the production and degradation of toxic proteins which accumulate in the brain causing neuronal death or can be differentially expressed because of cellular or tissue damages, aging and dysfunction of pro-survival proteins (24). Among PD-specific miRNAs, CSF and brain tissue of PD patients showed a significant down-regulation of miR1, miR331-5p, miR626, miR505, and miR19b-3p and the up-regulation of miR153, let7g-3p, miR409-3p and miR10a-5p. In the serum samples of PD cases, instead, increased amounts of miR30a/e and miR338-3p and lower expression of miR162-2-3p and miR1294 were observed (24, 25). Moreover, miR19b is one of the most interesting PD-associated miRNAs, since it has been found to be less expressed up to five years before the onset of motor symptoms. This result was reported in patients suffering from rapid eye movement and sleep disorders, which are common clinical features observed at the initial stages of different synocleinopathies, including PD. In fact, a 38% higher risk of progression has been estimated at 6 years of follow-up. In this context, miR19b has been proposed as predictive biomarker in patients affected by RBD, in which the conversion to PD is more likely to occur (25). Few studies tried to set-up panels of miRNAs able to discriminate PD cases from control subjects. Real Time PCR was the gold standard technique utilized for miRNA profiling, but the accuracy was not very high, with sensitivity and specificity values around 73–83% (25). In general, both serum and CSF proved to be good body fluids for developing proper miRNAs panels for patient's stratification, although CSF is known to allow a more efficient discrimination while serum requires less invasive procedures to be collected and is therefore less dangerous for patients (24). Several miRNAs have been also investigated for their modulatory activity on genes associated with neurodegenerative disorders (including PD). For example, miR7 and miR153 are known to inhibit *SNCA* expression while miR205 regulates *LRRK2*, which are known as two of the most implicated genes in PD etiopathogenesis (25). Over the association between miRNAs expression profile and the susceptibility to neurodegenerative disorders, increasing evidence proved that polymorphisms within the DNA sequence encoding the miRNAs can modify their expression and the affinity with the corresponding mRNA targets (26). These studies are the most recent approach utilized for clarifying the complex interactions occurring under physiological and pathological conditions and highlighted the existence of a “genetics of the epigenetics” contributing to the onset and progression of disease as well. This approach was applied to explore the genetics of epigenetics of Age-related Macular Degeneration (AMD), which is a neurodegenerative disease affecting the central portion of the retina and is highly frequent among people aged >65 years. This approach provided interesting insights into the pathogenesis and physiopathology of AMD, confirming that miRNAs represent one the most promising class of biomarkers with a prognostic, predictive, pharmacogenetic and clinical utility (26–28). In the case of PD, the interactions between polymorphisms of genes coding for miRNAs and the susceptibility to develop the disease are almost unknown. A study on a Chinese population detected a significant association between a SNP in *MIR4697* (rs329648, C/T, p = 8.21^*^10^−4^; OR = 1.87, 95%CI: 1.29–2.69) and the susceptibility to PD (29). Further investigation and replication studies on different populations are necessary to confirm this finding. Furthermore, it would be very interesting to provide a comprehensive knowledge of the genetics of epigenetics of PD as well as of other neurodegenerative disorders.

## Pharmacogenetics of PD

Large inter-individual variability is seen among patients treated with L-Dopa or other PD drugs in terms of drug response and adverse effects. In fact, the administration of these drugs has been associated with the occurrence of several chronic complications, including motor fluctuation (the drug effect does not last as long as expected), dyskinesia (uncontrolled movements), visual hallucination, psychotic reactions and sleep disturbances. LID is observed in 45% of subjects within 5 years of treatment, while approximately 25% of patients treated with dopaminergic agents experience hallucinations (30). Such a high inter-individual response to PD drugs could be explicated by the influence of specific genetic factors. Most of the knowledge about the pharmacogenomics of PD drugs deals with genes involved in dopaminergic activity, especially dopamine receptors (*DRD1, DRD2, DRD3*), transporters (*DAT, SLC22A1*/*OCT1*) and enzymes responsible for dopamine transformation and degradation (*COMT, MAO-B, DDC*) (14). In addition, genes involved in other neurotransmission pathways have also been found to influence the response to some PD drugs. The most investigated genes are *ANKK1, CCK, APOE, BDNF, ACE, MTHFR, HTR2A*, and *UGTA1* (14, 31). Among dopamine receptors genes, *DRD2* was the most investigated. On this subject, patients carrying the *DRD2* 13 and 14 repeat alleles (CA) of an intronic Short-Tandem Repeat (STR) were found to have a lower risk of LID, while carriers of *DRD2* 15 repeat allele displayed a better disease outcome (32). Interestingly, the SNP rs1800497 (C/T) is one of the most studied polymorphisms in PD and in response to therapies. It was initially mapped on *DRD2* gene but, later, it was demonstrated that it is located 10.5 kb from *DRD2*-3′ end, within the *ANKK1* gene. The T allele of rs1800497 has been associated with an increased risk of diskynesia and motor fluctuation in patients treated with L-Dopa; late hallucinations in case of treatment with L-Dopa and dopamine agonists, sleeps attacks following dopaminergic therapy. Moreover, rs1800497 may be in linkage disequilibrium with other variants of *DRD2* (such as−141C In/Del polymorphism) which have been associated with hallucinations after prolonged treatment of PD patients with L-Dopa (30, 32). Concerning the relationship between variants in *DRD3* and response to dopaminergic therapy, few studies highlighted the association of rs6280 (C/T, serine to a glycine amino acid change) with diphasic dyskinesia and visual hallucination after dopaminergic therapy (32). However, these data should be further investigated. A 40bp VNTR polymorphism (rs28363170) in 3′-UTR of *DAT1* gene has been found to be associated with a higher risk of dyskinesia, motor fluctuation and psychosis. Patients treated with L-Dopa and carrying the 9-repeats allele of the VNTR were found to have a 2.5 higher risk of developing dyskinesia. Similarly, the allele C of the rs393795 (A/C) in *DAT1* was found to increase the risk of dyskinesia; while the allele C of rs2652511 (C/T) was correlated to a higher prevalence of visual hallucinations in patients treated with dopaminergic drugs (31, 32). Several studies have also described the pharmacogenetic effect of variants within the *COMT* gene. In fact, the rs4680 (G/A, resulting in a Valine to Methionine amino acid change) appeared to strongly influence the response to L-Dopa treatment and the adverse effects upon administration of L-Dopa and other dopaminergic drugs (mainly entacapone) (14). In fact, carriers of the variant allele (A) of rs4680 may require a lower L-Dopa dose (less than 500mg after 5 years of treatment) with respect to those with the wild-type allele (G). On this subject, the analysis of a combination of 4 genetic variants of *COMT* gene (rs6269, A/G; rs4633, C/T; rs4818, C/G; rs4680, G/A) has been found to provide a better prediction of the response to L-Dopa. In presence of ACCG, ATCA and GCGG haplotypes, lower, intermediate and higher COMT activities have been described, respectively. The haplotype GCGG coding for the higher enzymatic activity has been associated with the need of a higher dose of L-Dopa. *COMT* haplotypes may therefore be highly helpful to prescribe individualized PD therapies in relation to patients' genetic profile (30). Moreover, the allele A of rs4680 has been associated with a higher risk of LID and sleep disturbances (excessive daytime sleepiness). The homozygous presence of the wild-type allele (G) of rs4680 (*COMT*) has also been associated with a better response to entacapone (a COMT-inhibitor drug), although this result needs to be confirmed on larger scale studies (32). Polymorphisms in *MTHFR* gene have been shown to affect the response to L-Dopa treatment. In particular, the rs1801133 SNP (C/T) has been found to influence the response to drug. The presence of the variant allele (T), especially at the homozygous state, has been described to increase the inhibition of COMT and prevent the development of resistance to L-Dopa (30). Finally, a number of studies have described significant associations between genetic variants and the increased risk of dyskinesia, hallucinations, sleeps attacks and psychosis upon treatment with L-Dopa and dopaminergic agents. These polymorphisms are mainly located in *APOE, CCK, ACE, BDNF, HOMER1* genes (30–32).

## The impact of non-genetic factors on neurodegenerative diseases

The previous sections of the review discussed the effects of the genotype and epigenotype on the neurological phenotype, especially concerning the predisposition to neurodegenerative disorders. As for epigenetics, even other exogenous, non-genetic factors can influence this predisposition without changing the DNA sequence. This is the case of nutrition, which is one of the main modulators able to determine optimal or suboptimal brain health throughout the lifetime. Nutrition participates in several processes related to neurodevelopment and neurogenesis which are essential for the maintenance of the homeostasis and plasticity of the central nervous system and the response to endogenous or exogenous stimuli (33, 34). In this context, nutrition and genes interact together to modulate such responses and protect the brain health. On the other hand, wrong or unbalanced diet can have a severe impact on brain function, contributing thereby to the onset and the worsening of disease. Growing evidence demonstrated the existence of a complex network of interactions among specific dietary components, genetic variants, epigenetic modifications (methylation pattern, miRNAs), environmental factors (such as stress and infections), age, sex, previous and current nutritional status which altogether determine the physiological or pathological functioning of the brain (33, 34). In this context, the Mediterranean diet has been extensively described as one of the most effective strategies to fight cognitive decline and neurodegeneration. In fact, it allows to take several nutrients with protective properties for the brain, such as polyphenols, Omega-3 fatty acids, zinc, copper, vitamins A, B, C, D and E, fish, fruits and vegetables (34). Polyphenols have been supposed to be involved in cognitive function and neurodegenerative disorders because of their antioxidant, iron-chelating properties and their activity in intracellular signaling pathways. Polyphenols are the most abundant antioxidant elements which can be drawn from multiple sources, including fruits, cocoa, tea, coffee and red wine (33). Among the different types of polyphenols, flavonoids have been extensively investigated because of their neuroprotective role against PD development. In fact, flavonoids have been shown to counteract the formation of tau proteins and the oxidative stress which are directly correlated with the onset of disease and neuronal death (35). In addition, a large-scale perspective study involving 130,000 subjects affected with PD reported that high flavonoid intake is associated with a 40% lower risk of developing the disease (35). Vitamins have also been described as possible adjuvant treatments in patients affected with neurodegenerative disorders, which often display low levels of folate, vitamin A, B12, C and E with respect to the healthy population (34). Vitamins supplementation has been demonstrated to delay or antagonize the neurodegenerative progression leading to PD. Regular intake of vitamin B6 has been associated with a lower risk of PD (*OR* = 0.65, 95% *CI* = 0.30–1.01). On the other hand, low intake of vitamin C and non-heme iron has been found to increase the risk of PD (*RR* = 1.92; 95% *CI* = 1.14–3.32). Indeed, iron metabolism has been supposed to be implicated in neurotoxicity and oxidative stress processes leading to the development of PD (34, 35). Omega-3 fatty acids are a type of polyunsaturated fatty acids (PUFs) which are mainly found in fish, vegetables oils, nuts, flax seeds and leafy vegetables. They are essential part of cell membranes ensuring the stability, fluidity, synaptic connectivity and avoiding the oxidative stress produced by ROS (34). Of the different kinds of PUFs, Docosahexaenoic Acid (DHA) and Eicosapentaenoic Acid (EPA) are the most investigated in neurodegenerative pathologies. Preliminary data hypothesized that PUFs may reduce the risk of PD (*RR* = 0.78; 95%CI:0.64–0.96), although further investigation is required to confirm and clarify this correlation. Interestingly, caffeine (contained in coffee, black or green tea and other beverages) has been extensively studied because of its neuroprotective effects against the onset of PD. After adjustment for possible confounding factors (such as smoking habit and age), the RR of PD has been estimated to be 0.72 (95% CI 0.65–0.81) considering a regular consumption of three cups of coffee/day (35, 36). Over nutrition, several studies tried to describe the positive or negative impact of smoking on the disease susceptibility and how to utilize this information into the clinical practice. To date, it is widely agreed that smoking during lifetime can influence the susceptibility to PD (36), especially by exerting a protective effect against the onset of disease. In fact, active and former smokers have been shown to have up to 74% lower risk of PD depending on dose and time of cessation (36). However, the biological significance of this association on disease neuropathology remains to be explained.

Among non-genetic factors, the possible correlation between physical/mental exercise and a higher predisposition to develop PD has also been investigated. The rationale for this hypothesis lies in the observation that dopaminergic neuronal loss and function improve with physical exercise in mouse models with PD. Supporting this thesis, three prospective studies reported a lower risk (*RR* = 0.7) of disease in men experiencing intense physical exercise with respect to moderate or recreational activities (36). However, these findings remain controversial and further work is necessary to understand the relationship between physical/mental exercise and the onset and progression of PD and other neurodegenerative disorders.

In conclusion, the data concerning the role of nutrition, smoking, physical/mental exercise in neurodegenerative disorders suggest that dietary and lifestyle interventions are helpful tools for preventing or modifying the course of PD. The impact of nutrition and smoking on brain health should be deeply investigated in relation to the inter-individual genetic variability which certainly impacts the response to specific foods and smoking-related compounds. In this perspective, personalized nutritional and lifestyle approaches may be adopted within the preventative and therapeutic programs currently in use for treating neurodegenerative pathologies.

## The microbiome and neurodegenerative disease

In recent years, the microbiome drew attention of a wide range of research groups, because of its strong impact on human health and disease. The Human Microbiome Project (HMP) characterized the microbial flora of 300 healthy individuals across different body sites (nasal passages, oral cavity, skin, gastrointestinal tract and urogenital tract). This study essentially highlighted a highly variable amount of microbial species across the human body, with the gut being the habitat with the highest concentration of microorganisms (approximately 100 trillion) (37). The HMP was extremely useful to find out that alterations of the gut microbiome can affect not only gastrointestinal diseases (inflammatory bowel disease, colon cancer) but also other systemic pathologies, such as allergy, diabetes, obesity, arthritis and cardiovascular disease (37). This association can be explained by a systemic influence of microbiome through a complex network of interactions including immune system, central nervous system (CNS), intercellular and signaling pathways. Special emphasis should be given to the bidirectional connection between gut and CNS, known as “microbiota-gut-brain axis.” In fact, if the effects of CNS on gut physiology have been proven over time, the possible impact of gut microbiota on CNS is now emerging (37, 38). Interestingly, gut microbial dysbiosis (unbalanced microbial populations) has been found to elicit the release of danger signals from CNS which ultimately lead to the activation of several pathways implicated in neurodegeneration, such as neuroinflammation, mitochondrial function, oxidative stress, microglial activation. In addition, the reduced microbiota diversity in the gut observed with aging has been shown to promote neurodegeneration and thereby the onset or the progression of neurodegenerative disease (38). Supporting this thesis, it is not surprising the higher prevalence of gastrointestinal-related comorbidities (diarrhea, vitamin deficiencies, constipation, obesity, diabetes) in patients suffering from neurodegenerative disorders (37). Moreover, some of the symptoms seen in the early phases of neurodegenerative pathologies, especially PD, occur at gastrointestinal level, suggesting that dysbiosis may be a triggering factor of disease and their management may be potentially used for preventative or treatment purposes (4, 37). PD patients showed a decreased prevalence of protective and anti-inflammatory bacterial species (*Prevotellaceae, Roseburia*, and *Faecalibacterium* spp.) together with a higher prevalence of proinflammatory groups (*Proteobacteria* spp.) and species associated with PD postural instability, worse motor function and fluctuations (*Helicobacter pylori* and *Enterobacteriaceae* spp.) (37, 38). Moreover, the accumulation of α-synuclein has been observed at level of the sigmoid mucosa of patients 2-5 years before the manifestation of neurological disorders. The authors of the study suggested that the α-synuclein is successively translocated to the brain through a prion-like manner or by inflammatory and oxidative stress processes (4, 37). Moreover, polymorphisms of the genes coding for peptidoglycan recognition proteins have been associated with PD risk in two independent studies, suggesting that these gene variants may impact the microbiota composition and the regulation of the immune response to commensal and harmful bacteria. As a result, gut mucosa would be more susceptible to inflammation, contributing thereby to the initiation of the neuropathological cascade through the accumulation of α-synuclein (4, 37). In addition, PD patients displayed lower levels of fecal Short-Chain Fatty Acids (SCFAs), which may be responsible for the reduced gastrointestinal motility and the disruption of intestinal homeostasis experienced by these patients (37). The microbiome has been found to affect the efficacy and the toxicity to drugs normally utilized for PD treatment. On the other hand, the medications could also alter the microbiome and cause adverse effects, as in case of patients taking COMT inhibitors which have been found to have an altered concentration of some microbial species (13). In this perspective, microbiome should be further investigated in order to understand its utility for drugs development or for enhancing treatment in a pharmacogenomic-like approach. Altogether, these findings show that manipulation of microbiome by administration of pro- and pre-biotics or microbial transplants could be helpful to alleviate PD-related symptoms, optimize drug response/toxicity, disease progression and improve quality of patient's life. The dissection of the complex interactions among microbiome, metabolic response, environment and host genome is still in its infancy and needs to be further investigated. However, microbiome certainly represents a powerful tool for a deeper understanding of neurodegenerative diseases, their progression and, subsequently, their management in the clinical practice.

## Conclusions

The application of precision medicine to the treatment and prevention of neurodegenerative disorders appears to be highly promising in contrast to the traditional “one-drug-fits-all” approach. In fact, neurodegenerative pathologies can present variable clinical features even in patients with the same disease who therefore are very unlikely to benefit of a single drug. In this context, the development of a precision medicine approach could represent an excellent possibility to identify preclinical stages of disease, make adequate differential diagnosis and provide timely and optimal treatments instead of the traditional treatments which are normally utilized at later stage of disease (11, 13, 39). The previous sections of the review highlighted how genes, neuroepigenetic modifiers, non-genetic factors (dietary, smoking habits, physical/mental exercise, microbiome) and drugs impact the susceptibility to neurodegenerative disorders. Prominent attention should be given to the dynamics of neuroepigenetic changes occurring at the inter- and intra-individual level. In fact, neuroepigenetic features are characterized by an elevated plasticity degree during the lifespan which ultimately modulate the function of specific genes in response to aging and specific environmental pressure, influencing thereby the likelihood to develop neurodegenerative disorders (40). The combination of the overall data can be utilized to generate “omic” profiles and provide a 360° overview of patients. The resulting “omic” profiles can be exploited by precision medicine to create a stratified medicine able to assign patients to specific treatment classes, such as high/intermediate/low risk individuals, good/intermediate/poor responders, high/intermediate/low dosage receivers. The translation of the theoretical concept of precision/stratified medicine into the clinical practice can be achieved by developing or even accommodating the computational facilities already in use to integrate the “omic” informations into a unique algorithm, able to predict a disease trajectory of patients and support the clinical and therapeutic decisions toward a more participatory and preventive medicine (40, 41). The availability of social networks for the simultaneous sharing of huge amounts of data across the world allowed to bridge the gap due to geographical distance and difficulties in getting access to these informations. In this context, the realization of a web-based network for neurodegenerative disorders can be decisive for the implementation of precision medicine strategies across different specialized centers. Such a network can contribute to accumulate and share the overall information derived from the combination of patient's participation (participative medicine) and the broad medical expertise provided by physicians. On this subject, an excellent example of multidisciplinary, web-based platform is the Italian IRCSS Network of Neuroscience and Neurorehabilitation, which is mainly addressed to standardize and optimize patient's clinical care and the therapeutic strategies applied to neurodegenerative disorders (42).

## Author contributions

CS: conception of the work, literature search, writing and revision of the manuscript drafts. VC and MG: writing of the first draft, literature search, manipulation of figures. SZ, GM, and SM: writing of the first draft and review of the final draft. RC and EG: conception of the work, revision and critique of the manuscript drafts.

### Conflict of interest statement

The authors declare that the research was conducted in the absence of any commercial or financial relationships that could be construed as a potential conflict of interest.

## Footnotes

^1^http://www.neurodegenerationresearch.eu

^2^http://www.pdgene.org, http://www.mdsgene.org/, http://www.alzgene.org/
